# Retrograde intubation in a dog with severe temporomandibular joint ankylosis: case report

**DOI:** 10.1186/s12917-018-1439-7

**Published:** 2018-03-27

**Authors:** Verónica Vieitez, Luis Javier Ezquerra, Víctor López Rámis, Massimo Santella, Ignacio Álvarez Gómez de Segura

**Affiliations:** 1 Veterinary Teaching Hospital, University of Extremadura, Avda, Universidad s/n, 10003 Cáceres, Spain; 2 Department of Animal Medicine and Surgery, Veterinary Faculty, University Complutense of Madrid, Avda. Puerta de Hierro, 28040 Madrid, Spain

**Keywords:** Dog, Difficult airway, Retrograde intubation

## Abstract

**Background:**

Orotracheal intubation in dogs is a common and easily-performed procedure that provides a patent airway during anaesthesia. In dogs with temporomandibular joint ankylosis or pseudo-ankylosis, airway management can be a challenging procedure since these dogs have a limited ability to open their mouth. Methods to provide safe, uneventful intubation in such patients may include minimally invasive techniques such as retrograde intubation using a guide wire and fibre-optic-aided laryngoscopy.

**Case presentation:**

We report a case of a 16-month-old, intact female Bull Terrier weighing 17 kg, admitted to the hospital for surgical treatment of bilateral ankylosis of the temporomandibular joint. Intubation was achieved, without direct observation of the larynx, by retrograde intubation using a vascular access catheter and a vascular wire guide through cricothyroid membrane. Bilateral condylectomy was performed and the dog recovered uneventfully.

**Conclusions:**

In conclusion, retrograde intubation was relatively simple to perform with the guide wire technique and no specific training or equipment were necessary.

## Background

Orotracheal intubation in dogs is a routine procedure, providing a patent airway through which to deliver oxygen and inhalant anaesthetic while facilitating ventilation and preventing aspiration of regurgitated material. Orotracheal intubation in dogs is an easily performed technique when using an adequately sized endotracheal tube (ETT) and adequate lighting. However, the use of a laryngoscope is always advised [1].

The “difficult airway” has traditionally been used to describe endotracheal intubation in patients with anatomic characteristics than make visualization of the vocal cords and placement of the endotracheal tube challenging [2]. The difficult airway is a rare condition in dogs, but in other species such as rabbits [3], camelids [4], pig [5] or mice [6], endotracheal intubation is considered technically difficult due to specific anatomical characteristics including size, a long, narrow oropharynx, large tongue, and limited mobility of temporomandibular joint (TMJ), which limit visualisation of the larynx.

Intubation in dogs with restricted mouth opening does not allow the use of laryngoscope and visualisation of the glottis. In people, a variety of pathological conditions such as TMJ ankylosis, oral submucosal fibrosis, and infection of the head and neck region are associated with reduction in mouth opening, making the intubation process difficult [7]. A variety of endotracheal intubation techniques have been advocated to manage the difficult airway, which include blind nasal intubation, retrograde intubation (RI), fibre-optic laryngoscopy and tracheostomy [8]. The RI of the trachea is an established airway management technique that can be used to place an ETT when more conventional methods (e.g. direct laryngoscopy) have failed [9].

The RI was reported in the early 1960s [10] as an alternative tool for unplanned preoperative tracheostomies in humans. This technique has evolved and is considered an approved method by the American Society of Anesthesiologists for the management of the difficult airways [11]. The elective use of RI is well-documented in patients with angioedema [12] deep neck infection [13], burns [14], trismus [15], musculoskeletal disorders [16], and patients in the prehospital or emergency room [17] and trauma setting [18]. In veterinary medicine, RI has been proposed as an alternative to conventional orotracheal intubation in mice [6], llamas [4], and rabbits [19]. Contraindications to the procedure include coagulation abnormalities, infection at the intended site of puncture, pre-tracheal mass and poorly palpable landmarks in the neck [18].

The relevance of this report relies on the fact that management of the difficult airway in dogs is scarcely documented [1]. Although flexible fibre-optic guided endotracheal intubation is the technique of choice for managing the difficult airway, its cost and availability of the device has limited its use. Therefore, RI is a practical alternative to manage these patients, also when fibre-optic guided technique fails [20]. The objective of this report was to describe a case in which RI was used electively in a dog with difficult airway due to severe TMJ ankylosis and limited mouth opening.

## Case presentation

A 16-month-old, intact female Bull Terrier weighing 17 kg was admitted to the hospital for the surgical treatment of bilateral ankylosis of the TMJ. The dog had been previously treated by the referring veterinarian with prednisolone (Dacortin 30 mg; Merck. Madrid, Spain) at a dose of 2 mg/kg orally once daily for 2 weeks. Clinical examination revealed a 4-mm mouth opening with minimal space between the maxillary and mandibular incisor teeth and atrophy of the temporal and masseter muscles. The results of physical examination, haematological and biochemical blood tests were unremarkable. A bilateral condylectomy was scheduled to treat the TMJ ankylosis.

Conventional orotracheal intubation was not considered, as direct visualization of the larynx would have been impossible due to the limited movement and opening of the TMJ. Therefore, RI was planned and the required equipment for RI and temporary tracheostomy were prepared.

Food, but not water, was withheld for 12 h prior to anaesthesia. A 20-gauge cephalic venous catheter (Insyte; Becton Dickinson. Singapore) was then aseptically placed. The dog was premedicated with intravenous acepromazine (Equipromacina; Labiana. Barcelona, Spain), 0.05 mg/kg and subcutaneous meperidine (Dolantina; Kern Pharma, Barcelona, Spain) 5 mg/kg, 30 min prior to induction. Following pre-oxygenation with 100% oxygen via a facemask (10 min), anaesthesia was induced with propofol (Vetofol; Esteve, Barcelona, Spain) titrated slowly to effect by administering initially as a bolus at 2 mg/kg over 30 s, followed by incremental doses at 0.5 mg/kg every 15 s with a total dose of 4 mg/kg. Then, a continuous rate infusion (CRI) of propofol (0.4 mg/kg/min) was started until the RI was performed (approximately 5 min later). Attempts at opening the mouth while the dog was anesthetized were unsuccessful and the maximum opening of the mouth only allowed the passage of a 6 mm (internal diameter, ID) endotracheal tube between the incisors.

**Fig. 1:**
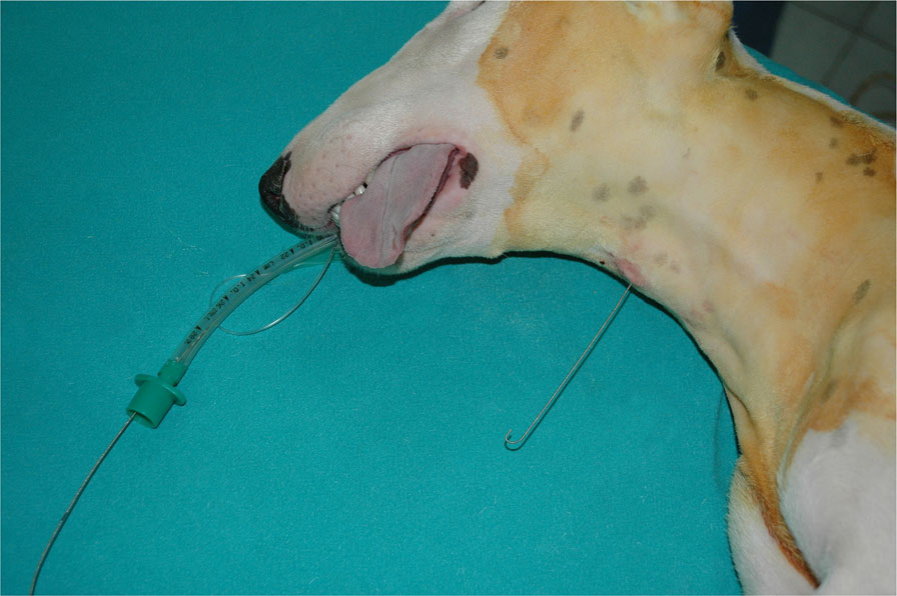
Lateral view of the dog. A vascular guide wire, advanced through the cricothyroid membrane and the larynx, can be observed exiting the mouth. The tip of the endotracheal tube is advanced over the wire guide through the mouth opening and advanced beyond the vocal cords into the larynx

For the RI technique, the dog was positioned in right lateral recumbency. The cricothyroid membrane was palpated and the skin over the trachea was clipped and aseptically prepared. A venous catheter 16-gauge (Insyte; Becton Dickinson. Singapore) and a Seldinger’s wire guide 0.8 mm diameter (Cook medical Limerick, Ireland) were used for RI technique. The anaesthetist was positioned in front of the dog and the larynx palpated and stabilized with the left hand while the cricothyroid membrane was punctured with a 16-gauge over-the-needle vascular access catheter. The needle was partially withdrawn into the catheter while it was advanced with a 45° angle in rostral direction. Tracheal placement of the catheter was confirmed by a lack of resistance to air aspiration through the catheter with a 6 mL syringe. The needle was removed and the wire guide was inserted throughout the catheter and advanced to the mouth opening. Then, the cannula was removed and the cricothyroid end of the wire guide was grasped with forceps to secure it. A cuffed ETT (6 mm ID) was passed caudally over the guide and into the larynx. Simultaneously, the mouth was forced open slightly to facilitate the tube passage between the incisive teeth. Once the ETT reached the entrance of the wire guide (previously measured) at the cricothyroid membrane, the wire guide was removed while slightly pushing the ETT to maintain its tip in the larynx. Then, the ETT was advanced into the trachea as the hold of the guide was removed (Fig. 1). Correct position of the ETT was confirmed using side-stream capnography (S/5 Anesthesia monitor: General Electric. WI, USA). The cuff was inflated immediately and the tube fixed.

Once intubated, the ETT was attached to the anaesthetic rebreathing system and mechanical ventilation was started using a pressure-controlled mechanical ventilator (Stephan ABC: Gackenbach, Germany). Peak inspiratory pressure (PIP) was set between 10 and 12 cm H_2_O, and f_R_ was adjusted to maintain normocapnia (partial pressure of expired carbon dioxide, PE’CO_2_ = 35-45 mmHg). The propofol infusion was stopped and anaesthesia was maintained with isoflurane (Isoflo 100%: Abbott. UK) delivered in 100% oxygen. Midazolam (Dormicum; Roche, Madrid, Spain) 0.5 mg/kg and fentanyl (Fentanest; Kern Pharma, Barcelona, Spain) 5 μg/kg were administered IV slowly over 60 s, followed by a CRI of midazolam (0.15 mg/kg/h) and fentanyl (6 μg/kg/h) administered IV throughout surgery using infusion pumps (Perfusor; BBraun, Germany).

Continuous electrocardiography, HR, f_R_, pulse oximetry (SpO_2_), PE’CO_2_, end-tidal isoflurane (F_E_’Iso) and inspired fraction of oxygen (F_I_O_2_) were monitored throughout anaesthesia using a multiparametric monitor (S/5 Anesthesia monitor: General Electric. WI, USA). Arterial blood pressure was measured noninvasively using a Doppler monitor (811-B; Parks Medical, OR, USA) with the probe placed on the metacarpal artery and fixed with tape.

After monitoring instruments were applied, left and right mandibular and maxillary nerve blocks were performed with the dog in lateral recumbency. After aseptic scrubbing of both lateral aspects of the face and neck, an extra-oral approach was used for left and right mandibular nerve block; a 22-gauge needle was inserted at the lower angle of the jaw approximately 0.5 cm rostral to the angular process and was advanced 2 cm dorsally along the medial surface of the ramus of the mandible. Bupivacaine (Bupivacaina 0.5%; Fresenius, Barcelona, Spain) 1 mL was injected blindly, because intraoral palpation of the mandibular foramen was not possible due to the limited mouth opening. For the maxillary nerve block, the needle was placed percutaneously along the ventral border of the zygomatic process approximately 0.5 cm caudal to the lateral canthus of the eye and advanced into close proximity of the pterygopalatine fossa. Bupivacaine 0.5% 1 mL was injected on each site. A total of 20 mg of bupivacaine (1.2 mg/kg) was administered.

Lactated Ringer’s solution (Ringer Lactato: B.Braun. Barcelona, Spain) was infused IV during surgery at 10 mL/kg/h. Cefazolin (Cefazolina: Normon. Madrid, Spain) 22 mg/kg was administered IV 30 min prior to surgical incision and then every 90 min throughout surgery. During surgery, F_E_’Iso ranged between 0.8 and 1.2%, HR between 77 and 84 beats/min, and systolic blood pressure between 90 and 120 mmHg. The SpO_2_ remained above 98% and PE’CO_2_ was between 35 and 45 mmHg.

The bilateral condylectomy was performed successfully. After completion of surgery, isoflurane delivery, midazolam and fentanyl CRIs were stopped. Morphine (Morfina 1%: Fresenius. Barcelona, Spain) 0.5 mg/kg and dexamethasone (0.5 mg/kg) IV were administered. Mechanical ventilation was maintained until attempts to breathe spontaneously were observed. The ETT remained in place until laryngeal reflexes returned 30 min after the isoflurane was discontinued. The dog had a quiet, calm recovery, with no signs of anxiety or dysphoria, and was transferred to a quiet, warm room. Total anaesthesia time (from anaesthetic induction to cessation of isoflurane delivery) lasted 170 min. Postoperative assessment of pain (Short Form of the Glasgow Composite Pain Scale) and physiological parameters were conducted hourly until discharge with no detected complications related to RI.

Postoperative analgesia was provided with morphine 0.5 mg/kg IV every 4-6 h as required for 24 h. Dexamethasone (Cortesona 2 mg mL^− 1^; Laboratorios Syva, León, Spain) 0.2 mg/kg was administered IV daily for 3 days. The dog was able to eat soft food, 4 h after extubation. Physical therapy 3-4 times daily included passive manually opening the jaw, holding it open for 5 s with moderate traction, and then closing the jaw. The diet was replaced with hard food immediately. One month after surgery the jaw opened correctly in an almost normal range.

## Discussion

Alternative methods to orotracheal intubation are more commonly used in species other than dogs. Nasotracheal intubation has been described as an alternative method of intubation in rabbits [3], foals [21], calves [22], and llamas [23]. The RI has been evaluated in mice [6], llamas [4], and rabbits [19] and, briefly, as an alternative method of intubation in dogs [1].

In the present case, RI was preferred compared to other techniques or methods for several reasons. The laryngeal mask is an effective, non-invasive method of securing an airway in dogs, cats, pigs and rabbits [24–27]. It has been employed to provide a patent airway in a brachycephalic dog with suspected masticatory myositis and trismus (2 cm of mouth opened) [28]. However, in our case, a smaller mouth opening prevented its use.

Fibre-optic-assisted nasotracheal intubation is the technique of choice for difficult airway management in people [29]. However, the cost of the equipment and the required skills and expertise necessary for its use makes the RI technique preferable in veterinary medicine [7]. The main advantage of this latter technique over the more commonly employed anterograde techniques of tracheal intubation is that the laryngeal inlet does not have to be identified. It is a minimally invasive, feasible, and cost effective airway management technique in people with restricted mouth opening [7]. Unlike fibre-optic bronchoscope-guided endotracheal intubation, RI can be used when blood or secretions are present in the upper airway, where the former technique is difficult or impossible [30]. In this case, a flexible bronchoscopy device would have been an alternative for endotracheal intubation, but bronchoscope small enough to fit a 6 mm ETT is required and was not available.

A blind insertion airway device, the double lumen airway device (Combitube), has been evaluated in dogs [31] to provide ventilatory support. However, due to the inability to consistently seal the airway, the caudal aspect of the mouth is not fully protected for maxillofacial procedures including mandibular resection [31]. Additionally, the size of the device (11.1-12.1 mm in diameter) should have prevented its use.

In human another alternative method is fluoroscopic assisted airway intubation [32] using a nasal approach. Nasotracheal intubation has not been described in dogs, probably due to the small size of the nasal airways, which makes intubation traumatic or impossible. Also fluoroscopic guidance would unnecessarily expose personnel to radiation. Therefore, after considering all potential alternatives, RI was considered the most suitable way to establish and maintain a patent airway while avoiding tracheostomy.

The RI can be performed with minimal equipment. Although the RI set (Retrograde Intubation Set: Cook, Bloomington, IN, USA) is preferable, a standard venous catheter and epidural catheter or Seldinger’s wire can be used for retrograde technique. This allows the technique to be employed by almost any clinical practice [7].

There are some differences in RI between humans and animals. In rabbits, the trachea is small, movable and more difficult to puncture and may require surgical exposure to insert the guide via direct puncture of the tracheal wall [19]. In llamas, incision of the skin over the trachea and between two tracheal rings has been performed [4]. However in humans like in dogs, the cricothyroid membrane is easy to localize and puncture, facilitating the introduction of the guide. Although the short distance between the cricothyroid membrane and the vocal cords allows the unattached wire to inadvertently be withdrawn above the cords, thereby losing tracheal access and increasing the risk of failed intubation [9]. In humans, the insertion of a catheter into the trachea before the removal of the guide wire may help to cope with this problem [20]. Anterograde over a retrograde guide may improve the efficacy of RI and is considered to be a more reliable and preferable technique for improving the success rate [33] without prolonging its duration [20].

In human medicine the RI encompasses several methods of translaryngeal guided non-surgical airway access to facilitate orotracheal or nasotracheal intubation. It has been used in awake, obtunded or apneic human patients when other methods have been unsuccessful, unavailable or contraindicated [34]. In an effort to make the technique more reliable and easier to perform, RI has undergone numerous modifications. Significant improvements include the Water’s technique, which uses an epidural catheter to guide the ETT [35], the use of the Murphy’s eye as a conduit for retrograde wire [36], and the pairing of the RI technique with other airway management techniques in an effort to increase the success of the procedure included the use of a fiberoptic bronchoscope [37], a light wand [38], a Combitube [39], a modified Eschmann stylet [40], a laryngeal mask airway [41], a Mini-Trach II kit [42], a Cook airway exchange catheter [43], a gastric tube [44], and even a Fogarty embolectomy catheter [38].

Although RI is reported as a simple and reliable method that can be performed easily and quickly in experienced hands, it has also been associated with a low success rate and a significant risk of complications [45]. In human medicine complications of RI are few; the most common is a sore throat, affecting 60% of the patients [45]. More severe complications are rare and the most common include hypoxemia, cough, laryngospasm/bronchospasm [46], development of hematoma, incorrect site of tube placement, procedure time greater than or equal to 3 min, unsuccessful tube placement, laryngeal fracture with permanent dystonia [33], pneumomediastinum [20], subcutaneous emphysema [47], infection, and bleeding [48].

In a recent retrospective report of 20 patients with restricted mouth opening receiving RI, three patients developed sore throat and cough after intubation, one bronchospasm, and another patient developed wound infection [7]. There are no reports of complications related to this technique in rabbits [19], mice [6], or llamas [4]. In the latter species, RI was evaluated as an alternative to traditional intubation with fewer attempts needed to place the tube and no relevant side effects were observed. Clinicians familiar with camelid and goat intubations considered the RI technique faster and easier, requiring fewer personnel compared with the traditional technique [4].

## Conclusion

In conclusion, a dog with severe TMJ ankylosis and limited mouth opening was successfully intubated by RI without complications. The RI technique was considered safe, and minimally invasive, simple to perform and no specific training or equipment was required. Therefore, although new intubation techniques and devices have been developed, the RI remains useful in patients requiring blind orotracheal intubation due to inadequate mouth opening.

## Abbreviations

CRI: Continuous rate infusion
ETT: Endotracheal tube
F_E_’Iso: End-tidal isoflurane
F_I_O_2_: Inspired fraction of oxygen
f_R_: Respiratory rate
HR: Heart rate
LM: Laryngeal mask
PE’CO_2_: Expired carbon dioxide
PIP: Peak inspiratory pressure
RI: Retrograde intubation
SpO_2_: Haemoglobin oxygen saturation
TMJ: Temporomandibular joint
